# The impact of revitalized urban and rural recreation infrastructure on usage levels: evidence from a longitudinal quasi-experimental study in Alberta, Canada

**DOI:** 10.24095/hpcdp.46.3.03

**Published:** 2026-03

**Authors:** Ana Paula Belon, Laura Nieuwendyk, Vijaya Krishnan, Candace I.J. Nykiforuk

**Affiliations:** 1 School of Public Health, University of Alberta, Edmonton, Alberta, Canada

**Keywords:** natural experiment, surveys and questionnaires, physical activity, sports and recreational facilities, parks

## Abstract

**Introduction:**

Few studies have analyzed the impact of public investments in indoor and outdoor recreational spaces, and even fewer have assessed this impact longitudinally. This hinders informed decision-making about returns on investments made with limited public budgets. We assessed the impact of a 2008 municipal plan to revitalize existing urban and rural public indoor facilities and outdoor spaces by evaluating changes in usage levels before and after implementation of Phase 1 (2009–2013) of the revitalization plan.

**Methods:**

A quasi-experimental study involving a telephone survey of 750 participants was conducted before and after Phase 1. A region with similar demographics and public recreational indoor and outdoor infrastructure was used for comparison.

**Results:**

Our analysis found no changes in usage of recreational venues over time whether indoor (e.g. multipurpose recreational facilities, community halls) or outdoor (e.g. golf courses, off-leash dog parks, multiuse trails), in either the intervention or comparison region. Only one rural multipurpose indoor recreational facility showed a statistically significant increase in usage during Phase 1.

**Conclusion:**

Strategies targeting only physical infrastructure may not result in increased usage across a municipal population. To address existing inequities in access to publicly funded community resources that support health, both the built and social environments must be considered.

**Highlights**

- Using longitudinal data in a quasiexperimental study, we found that despite investment in revitalizing existing public urban and rural indoor recreational facilities and outdoor spaces, usage increased in only one rural indoor multipurpose facility.
- Upgrades to physical infrastructure may not have addressed barriers to usage, for example, a lack of diverse programming or high fees.
- Addressing residents’ perceptions and experiences may be critical to ensuring that they increase their use upgraded public recreational facilities more.

## Introduction

Access to public indoor recreational facilities and outdoor spaces can increase population levels of physical activity (PA), promoting mental and social health^1^-^5^ and preventing chronic diseases.^2^,^6^ There is also strong evidence for the cost-effectiveness of environmental interventions in increasing facility usage and PA levels.^7^-^9^

Given its critical role in health and well-being promotion, public PA infrastructure should be primed to meet the evolving community needs that come with population growth and aging, changes in ethnic composition, and so on. However, construction of new recreational spaces may be unfeasible environmentally or financially, including in municipalities operating with smaller budgets. Revitalizing existing infrastructure may be a more viable way to invest in the longer-term health of the community. Municipal revitalization projects can attract new users while helping current users maintain or increase their facility usage.^10^,^11^ An indirect, expected outcome of such revitalization is the creation of more vibrant communities with increased everyday social interactions.^11^,^12^ Public infrastructure also offers recreation opportunities at lower or no cost to the entire socioeconomic spectrum.^1^,^11^

There are few studies on the impact of environmental interventions that promote the usage of public recreational venues and even fewer that assess such impact longitudinally.^1^ Most natural or quasi-experimental assessments of recreational venues are conducted within one year of their revitalization^13^ and use direct observation or user surveys.^1^ Studies with short follow-ups and smaller sample sizes are more likely to report null or mixed results.^1^,^13^ Evidence for effective environmental interventions is clearly needed^7^ to better support government strategic planning and resource allocation. Further, there is a need for research that focuses on rural settings;^14^ these settings often lack PA infrastructure (or have to deal with the maintenance of aging facilities), leading to widening rural–urban inequities in leisure-time PA engagement.^14^,^15^

The implementation of a municipal plan for the revitalization of existing urban and rural recreational venues in a mid-sized Canadian municipality served as an opportunity for a quasi-experimental study to assess impact on usage levels and, ultimately, identify the value of such investment. We evaluated the difference in usage of this infrastructure from before the implementation of the revitalization plan to after its implementation (2009–2013) in that region, compared to a proximal region of similar size and profile.

***The municipal revitalization plan***

In response to demographic changes and community demands for more recreation infrastructure, the intervention region (in Alberta, Canada) launched a strategy to revitalize public recreational spaces in 2008. As a result of extensive public engagement and consultation, the 15-year strategic plan (2009–2023) was divided into three 5-year phases. After conducting needs and impact assessments (including cost–benefit and environmental impact analyses), the region opted to expand and improve three indoor venues at a lower cost instead of constructing a new multipurpose indoor recreational facility.^16^ Investment in 13 outdoor spaces included improvement of the trail network, landscaping, baseball diamond revitalization or improvement, new outdoor skating surfaces, a new bike skills park, new playgrounds and acquisition of park furniture and signage in the urban and rural areas within this region.^16^ The aim was to create an array of interconnected recreation opportunities across major and specialized indoor and outdoor spaces in order to promote use and improve residents’ physical health and well-being. The variety of revitalized recreational venues across the region would suit residents’ preferences and meet their needs for diverse activities and sports.

This study evaluated the impact of Phase 1 of the 15-year strategic plan to revitalize public recreational spaces. 

***Settings***

The intervention region is a “specialized municipality,”^17^-^19^ a municipal structure that is made up of small settlements (in this case, nine hamlets) with a total area of 1170.65 km^2^. One of these hamlets is urban (the largest in Alberta) and eight are rural. To assess the trends in the intervention region, we used a comparison region comprising three municipalities (totalling 2448.64 km^2^)^18^: a city (City of Spruce Grove), a town (Town of Stony Plain) and a municipal district (Parkland County) that includes rural areas such as hamlets, unincorporated communities and summer villages. These three municipalities are referred to as the “tri-municipal region” as they are near to each other and work closely together.

We chose this tri-municipal region as the comparison region because of the numerous similarities with the intervention region in terms of demographics, PA infrastructure, geographic location and climate. More specifically, in 2011 the intervention and comparison regions had populations of 92403^20^ and 71790,^21^ respectively, and population growth rates higher than the national average of 5.9%,^20^ at 12.1%^20^ and 17.4%.^21^ Each region had one multipurpose indoor recreational facility, a small number of specialized indoor facilities and a variety of outdoor recreational spaces in urban or rural areas; these facilities were owned and operated by the municipalities.^22^ Another deciding factor was that both the intervention and comparison regions are within 30 km of Alberta’s capital, Edmonton, but sufficiently far apart from each other to limit study contamination, for example, by residents’ using the other region’s facilities.

Despite their similarities, the regions were at different stages of their recreational facility policies and funding opportunities at the time of the study. After public consultation in 2009, the comparison region developed a business plan that guided investment in existing recreation infrastructure with a shared budget of CAD 105000, which was approved in 2015. The intervention region’s budget was CAD41 million for Phase 1. None of the venues were closed during their upgrading. Table 1 shows an overview of the indoor recreation facilities and outdoor spaces in the intervention region that underwent upgrading.

**Table 1:** Revitalization of each indoor facility and outdoor space owned and operated by the municipal government in the intervention region, Alberta, Canada

Recreational space / location^a^ / access	Description	Revitalization activities (completed between 2011 and 2013)
Indoor		
Multipurpose recreational facility Rural Fee-based	Includes a fitness centre, 2 ice-skating areas, an indoor track, a curling rink, a play-based room for preschool-aged children, an indoor playground, a team-training room, a fitness studio and a youth lounge	Renovated 1 full-size arena, built 1 new full-size indoor arena with an ice sheet and added a 557 m^2^ fitness centre, a new indoor track, community spaces, an indoor playground and multipurpose rooms
Arena (ice and dry surface) Urban Cost-free and fee-based	An ice arena (two-thirds of a regular size) with dry surfaces in the spring and summer	Renovated and retrofitted the ice arena
Multipurpose recreational facility Urban Fee-based	The largest multipurpose recreation centre in the region includes a wellness centre, an aquatics centre, 2 ice arenas, a leisure ice surface, a gymnasium, 2 indoor soccer fields and an indoor playground	Doubled the size of the indoor leisure ice surface, added to the wellness centre and increased track accessibility; built a new group fitness space and a new youth lounge; expanded the gymnasium and the indoor playground; and added multipurpose community activity rooms
Outdoor		
Golf course Urban Fee-based (in the summer)	An 18-hole golf course	Renovated the indoor community clubhouse
Off-leash dog parks Urban and rural Cost-free	Large off-leash dog parks with fenced areas, enclosed spaces for smaller dogs and boarded rinks	Improved trails and open areas inside the dog parks and added new park furniture and signage
Parks, playgrounds and green spaces Urban and rural Cost-free	The many different parks and playgrounds include amenities such as basketball hoops, firepits and a trail system connecting ponds. Most parks are wheelchair accessible and include specialized equipment	Upgrades at various locations included the addition of a community garden space, a spray deck, playgrounds, seating, gazebos, tree plantings, a viewing deck, interpretive signage, trails around the wetlands and, whenever possible, trail access points Within the BMX/bike skills park, a children’s pump track was added alongside dirt ramps, rock boulders and logs, which together make ladder bridges, a wall ride and different-sized dirt jumps for all ages and skill sets
Community ball diamonds Urban and rural Cost-free	More than 40 community baseball diamonds, of which 9 are Class A premier, i.e. high-quality amenities requiring more frequent maintenance	Four baseball diamonds with expanded infields and outfields, new sod and foul-line fencing, backstops, dugouts and granular trails
Outdoor skating surfaces Urban and rural Cost-free	More than 20 outdoor skating surfaces that are open between December and March (weather dependent). Most have lights, boards and skating pathways	A new outdoor ice rink with boards, lights, newly planted trees and park furniture. A relocated leisure ice-skating area
Multiuse trails Urban and rural Cost-free	More than 200 km of trail (asphalt, gravel, shale or limestone)	New and upgraded trails with 3 m wide asphalt paving and connections with other trail systems

^a^ Any areas outside of population centres, which have populations of at least 1000 people and population densities of at least 400 people per km^2^, are considered rural areas in Canada.^23^ In the intervention region, rural areas include hamlet communities, residential acreages and large and small lots for agricultural operations. 

The intervention region’s department of recreation, parks and culture and the three municipalities comprising the comparison region partnered with our research team for this quasi-experimental study. Partners informed the study design, contributed to the development of data collection tools and helped to interpret data using their in-depth knowledge of community-specific information. 

The study received ethical clearance from the University of Alberta Health Research Ethics Board (Pro00022189).

## Methods

This mixed-methods quasi-experimental project involved telephone surveys and focus groups before and after Phase 1, and the revitalization of public recreational spaces to increase usage, in both regions. The telephone survey includes longitudinal and repeated cross-sectional designs. In this article, we analyze longitudinal data obtained through the telephone surveys. Data obtained from the focus groups are published elsewhere.^24^

***Data collection***

Two digital dial telephone surveys were conducted before (August to October 2011, i.e. end of summer to mid-fall) and after (August to October 2013, i.e. end of summer to mid-fall) the Phase 1 implementation. 

For the 2011 survey, participants were recruited by a contracted survey firm that randomly selected residents’ telephone numbers. Potential participants were asked if they were 13 years or older and resided in one of the municipalities in each region; they were also asked the first three digits of their postal code. Awareness or previous use of either the indoor or outdoor recreational facilities and spaces were not eligibility criteria. 

The survey firm called residents up to five times over a 2-week period and requested the participation of the household member with the birthday coming next to facilitate stratification of respondents by age category and sex. Targeted recruitment of youth aged 13 to 17 years and adults aged 18 to 29 years was undertaken to reach these population segments. On calling a household, the survey firm asked if there were any people in those two age groups living in the household; if yes, the interviewer asked if they could talk to them and invited them to participate in the telephone survey. 

After completing the survey, participants had the option to provide their contact information if they wished to participate in a follow-up survey. They were subsequently contacted to confirm their interest in continuing to participate. New participants were also randomly selected in 2013 to mitigate attrition since 2011 and to increase the sample size to permit a more robust analysis (reported elsewhere^25^,^26^). 

The pre-intervention survey had 1045 and 1047 participants in the intervention and comparison regions, respectively, and the post-intervention survey 1057 and 1045 participants. Of these, 64 participants from the intervention region and 69 from the comparison region were removed because of self-reported inconsistencies in age and sex. We analyzed the participant populations in both surveys—406 in the intervention region and 344 in the comparison region, to a total of 750, or 38.2% of all 2011 participants who responded to the 2013 questionnaire.

The questionnaires collected data on demographics, self-reported health, self-reported weekly PA engagement and attitude toward and use of recreation infrastructure. Using a validated questionnaire,^27^,^28^ we asked participants about their levels of leisure-time PA in the past week. Attitudinal variables were based on agreement (on a scale from 1, for “strongly disagree,” to 10, for “strongly agree”) with the following four statements: “I would use indoor public recreational facilities more often if it weren’t for personal reasons”; “I would use indoor public recreational facilities more often if the facilities better met my needs”; “I would use outdoor public recreational facilities more often if it weren’t for personal reasons”; and “I would use outdoor public recreational facilities more often if the facilities better met my needs.” The expressions “personal reasons” and “met my needs” were left undefined and the respondents were free to interpret these as they wished. 

We also asked participants about their use of each venue in the last year. The response options were 0 for “never,” 1 for “less than monthly,” 2 for “monthly,” 3 for “weekly” and 4 for “daily.”

The list of the facilities and outdoor spaces revitalized in Phase 1 was provided by the intervention region prior to the survey. Only those facilities and spaces where revitalization had been completed by 2013 were included in the 2013 survey questionnaire. Given that the revitalized spaces were in both urban and rural areas across the region, most of residents could have benefitted from the upgrades.

***Data analysis***

Baseline characteristics of both regions were compared through chi-square tests and *t*tests. Item nonresponse was the main source of missing survey data. Approximately 30% of responses for annual household income were missing in each region; to address this, we imputed a median income of CAD70000, the median household income in Canada at the time of the study.^29^ Tests were performed to ensure data were not skewed with the imputation but remained representative of the populations in each region. That classification was used to distinguish households with an annual income of CAD70000 or less versus more than CAD70000. 

Following the guidelines suggested by Godin,^28^ participants’ answers to mild, moderate and strenuous PA weekly engagement were multiplied by 3, 5 and 9, respectively. A total score was calculated based on the sum of the products, which was classified into physically active (scores ≥24 units) or moderately/insufficiently active.

We analyzed the trends in public PA infrastructure usage using paired *t* tests. For comparative analyses, we selected indoor and outdoor spaces in the comparison region to match revitalized ones in the intervention region. Four summary indexes compiling usage data were created based on frequency of use for the indoor facilities (three in the intervention region and five in the comparison region) and outdoor spaces (six in both regions).

Given the study’s time span, a two-way analysis of covariance (ANCOVA) was conducted on all indoor and outdoor spaces with statistically significant changes in usage over time. We analyzed whether variables showing significant differences between the two regions in 2011 influenced the change over time (main and interaction effects). A number of assumptions associated with ANCOVA were tested before applying the technique to ensure no violation of normality, reliability of measurement of the covariate, linearity, homogeneity of variances or homogeneity of regression slopes.^30^ We calculated eta-squared values for all indoor and outdoor spaces with significant changes over time to assess the magnitude of the revitalization effect.

Of note, a sensitivity analysis comparing the characteristics of the sample of participants who responded to both surveys (n=750) and the sample of participants who only completed the 2011 survey (n=1209, after removal of the 133 cases with inconsistent data on age and sex) detected statistically significant differences. The sample of participants who completed both surveys was more educated (*p*=0.001), included fewer full-time workers (*p*=0.004), reported better self-rated health (*p*=0.013) and were more likely to agree that indoor facilities met their needs (*p*=0.034).

## Results

Relative to the comparison region, the intervention region had a higher proportion of urban residents, higher usage of indoor facilities, better self-rated health, lower usage of outdoor spaces and less agreement that the indoor and outdoor spaces met residents’ needs (Table 2).

**Table 2:** Baseline characteristics of study populations in the intervention and comparison regions, Alberta, Canada, 2011 and 2013

Variables	Total (n = 750)	Intervention region (n = 406)	Comparison region (n = 344)	*p* value^a^
**Mean age, years**	48.9	48.4	49.6	0.347
Sex, %				
Male	41.5	40.9	42.2	0.726
Female	58.5	59.1	57.9	0.726
Area of residence, %				
Rural	34.5	27.6	42.7	0.000
Urban	65.5	72.4	57.3	0.000
Education level, %				
Postsecondary graduate	76.2	79.0	73.0	0.058
Less than postsecondary	23.8	21.0	27.0	0.058
Employment, %				
Part-time/retired/other	60.2	60.7	59.6	0.765
Full-time	39.8	39.3	40.4	0.765
Annual household income, %				
< $70 000	52.3	50.0	54.9	0.177
≥ $70 000	47.7	50.0	45.1	0.177
Household size and composition				
0 children in the household, %	58.8	58.4	59.3	0.797
≥ 1 children in the household, %	41.2	41.6	40.7	0.797
Mean household size, n	3.0	3.1	2.9	0.319
Leisure-time physical activity				
**Mean total weekly score^b^**	48.3	51.0	45.6	0.405
Weekly score, %				
Moderately or insufficiently active participants (< 24)	23.9	22.7	28.3	0.076
Physically active participants (≥ 24)	76.1	77.3	71.7	0.076
Self-rated health, %				
Low (poor/fair/good)	39.2	31.6	48.3	0.000
High (very good/excellent)	60.8	68.4	51.8	0.000
Mean frequency of use and meeting usage needs				
Frequency of use of indoor facilities^b^	2.1	2.2	1.9	0.002
Frequency of use of outdoor spaces^b^	2.8	2.6	2.8	0.050
Meeting indoor usage needs	5.5	5.0	6.0	0.000
Meeting outdoor usage needs	4.4	4.2	4.7	0.009

^a^ *p* values are based on the chi-square test of statistical independence for categorical data and the Student *t* test for parametric data. 

^b^ Weighted mean. 

In the intervention region, usage of the rural indoor multipurpose recreational facility increased (Table 3), but the golf course recorded a decrease in usage. In the comparison region, the usage of the golf course and off-leash dog parks decreased over time. The eta-squared values (0.40 for rural indoor multipurpose recreational facility and 0.30 for golf course in the intervention region; 0.49 for golf course and 0.29 for off-leash dogs park in the comparison region; data not shown) indicate large effect sizes^31^ for the significant differences in the usage. 

**Table 3:** Usage of each indoor and outdoor facility in the intervention and comparison regions, Alberta, Canada, 2011 and 2013 (n = 750 participants)

Recreational spaces	n^a^	Usage mean^b^				*p* value	95% CL
		2011	2013	Change	SD		
Indoor							
Intervention region							
Rural indoor multipurpose recreational facility	52	0.88	1.42	0.54	1.26	0.003	0.19, 0.99
Urban indoor arena (ice and dry surface)	41	0.98	1.17	0.20	1.10	0.253	−0.15, 0.54
Urban indoor multipurpose recreational facility	264	1.98	1.89	−0.09	1.18	0.231	−0.23, 0.06
Comparison region							
Urban indoor multipurpose recreational facility	221	1.95	1.83	−0.11	1.04	0.107	−0.25, 0.02
Urban indoor arena (ice and dry surface)	32	1.31	1.13	−0.19	1.15	0.363	−0.60, 0.23
Urban indoor arena (ice and dry surface)	78	1.35	1.17	−0.18	1.04	0.132	−0.41, 0.06
Rural indoor community halls	36	1.31	1.36	0.06	1.31	0.800	−0.39, 0.50
Urban indoor community halls	48	1.27	1.63	0.35	1.42	0.091	−0.06, 0.77
Outdoor							
Intervention region							
Golf courses	59	1.58	1.32	−0.25	0.82	0.021	−0.47, −0.04
Off-leash dog parks	36	2.47	2.28	−0.19	0.86	0.182	−0.48, 0.10
Parks, playgrounds and green spaces	242	2.68	2.53	−0.15	1.28	0.073	−0.31, −0.04
Community ball diamonds	47	1.79	1.74	−0.04	1.27	0.819	−0.41, 0.33
Outdoor skating surfaces	81	1.93	1.98	0.05	1.13	0.695	−0.20, 0.30
Multiuse trails	61	2.38	2.54	0.16	1.13	0.261	−0.12, 0.45
Comparison region							
Golf course	34	1.85	1.53	−0.32	0.59	0.003	−0.53, −0.12
Off-leash dog parks	47	2.40	2.11	−0.30	0.10	0.047	−0.59, −0.01
Parks, playgrounds and green spaces^c^	214	2.33	2.18	−0.15	1.39	0.116	−0.15, −0.34
Community ball diamonds	34	1.65	1.44	−0.21	1.16	0.314	−0.62, 0.20
Outdoor skating surfaces	82	1.66	1.76	0.01	0.83	0.288	−0.08, 0.28
Multiuse trails	245	2.00	1.98	−0.03	1.16	0.741	−0.17, 0.12

**Abbreviations: **CL, confidence limit; SD, standard deviation. 

**Note:** Paired t tests were performed for within-group comparisons. 

^a^ Number of people who reported using the facility. 

^b^ Based on responses on a 5-point scale where 0 stands for “never,” 1 for “less than monthly,” 2 for “monthly,” 3 for “weekly” and 4 for “daily.” 

^c^ Refers to a composite variable. 

Residents in both regions reported monthly usage of the indoor facilities (mean= 1.98–2.04) and weekly usage (mean=2.57–2.71) of the outdoor spaces in 2011 and 2013 (Table 4). The results indicated no significant change in any of the summary indexes for indoor and outdoor venues in both regions between 2011 and 2013.

**Table 4:** Usage of all indoor and outdoor facilities in the intervention and comparison regions, Alberta, Canada, 2011 and 2013

Recreational spaces	n^a^	Usage mean^b^				*p *value	95% CL
		2011	2013	Change	SD		
Intervention region							
Overall indoor usage^c^	276	1.98	1.98	0.00	1.23	1.000	−0.15, 0.15
Overall outdoor usage^d^	282	2.71	2.62	−0.09	1.19	0.232	−0.22, 0.05
Comparison region							
Overall indoor usage^c^	230	2.04	2.00	−0.04	1.14	0.564	−0.19, 0.10
Overall outdoor usage^d^	246	2.69	2.57	−0.12	1.15	0.099	−0.27, 0.02

**Abbreviations: **CL, confidence limit; SD, standard deviation. 

^a^ Number of people who reported using the facility. 

^b^ Based on responses on a 5-point scale where 0 stands for “never,” 1 for “less than monthly,” 2 for “monthly,” 3 for “weekly” and 4 for “daily.” 

^c^ Monthly usage. 

^d^ Weekly usage. 

For the two-way ANCOVA, we included those variables with significant differences in 2011 (Table 2). Agreement with meeting indoor and outdoor needs, despite differences, was not included because of violation of the assumption on homogeneity of slopes (Table 5). 

**Table 5:** Results of two-way ANCOVA: tests of between-subject effects

Recreational spaces	Type III sum of squares	*F* statistic	*p *value	Partial eta-squared	Adjusted *R^2^*
Intervention region					
Major rural indoor multipurpose recreational facility					
Adjusted model	16.152	3.755	0.010	0.242	0.178
Area of residence in 2011	5.045	4.691	0.035	0.091	0.178
Self-rated health in 2011	5.116	4.758	0.034	0.092	0.178
Frequency of use in 2011	2.295	2.135	0.151	0.043	0.178
Area of residence in 2011 self-rated health in 2011	0.049	0.460	0.831	0.001	0.178
Golf course					
Adjusted model	13.128	6.696	0.000	0.336	0.286
Frequency of use in 2011	12.488	25.480	0.000	0.325	0.286
Area of residence in 2011	0.000	0.000	0.985	0.000	0.286
Self-rated health in 2011	0.435	0.888	0.350	0.160	0.286
Area of residence in 2011 self-rated health in 2011	0.534	1.089	0.301	0.200	0.286
Comparison region					
Golf course					
Adjusted model	15.419	15.852	0.000	0.686	0.643
Frequency of use in 2011	14.025	57.676	0.000	0.665	0.643
Area of residence in 2011	0.163	0.669	0.420	0.023	0.643
Self-rated health in 2011	0.068	0.278	0.602	0.010	0.643
Area of residence in 2011 self-rated health in 2011	0.068	0.278	0.602	0.010	0.643
Off-leash dog parks					
Adjusted model	17.377	5.514	0.001	0.344	0.282
Frequency of use in 2011	11.974	15.197	0.000	0.266	0.282
Area of residence in 2011	3.418	4.338	0.043	0.094	0.282
Self-rated health in 2011	0.503	0.638	0.43	0.015	0.282
Area of residence in 2011 self-rated health in 2011	1.326	1.683	0.20	0.039	0.282

**Abbreviations: **ANCOVA, analysis of covariance; R^2^, multivariate coefficient of determination. 

Overall, all four models were significant, with large effect sizes. For the rural indoor multipurpose recreational facility, effects of area of residence and self-rated health on the increased usage over time were statistically significant with moderate effect sizes. The results suggested no interaction effect from the two independent variables. The 2011 usage had a statistically significant and large effect in the model of the intervention region’s golf course. Neither of the main nor the interaction effects of residence and self-rated health were significant.

For the comparison region’s golf course model, significant and large effects were found in the 2011 usage. For the off-leash dog parks in the comparison region, the 2011 usage of these outdoor spaces and area of residence had statistically significant effects. The effect sizes were large for frequency of use in 2011 and moderate for area of residence.

## Discussion

This quasi-experimental study showed that Phase 1 of a 15-year municipal plan to revitalize urban and rural recreational indoor and outdoor spaces did not increase usage levels in most of the renovated venues over a 2-year period. After a new arena for recreational skating, a wellness centre, a fitness track and an indoor playground were built, usage of the rural multipurpose indoor recreational facility increased. 

While the lack of indoor recreational facilities in rural areas in North America is an issue,^14^,^15^ the existing infrastructure in the intervention region may have been obsolete, not meeting people’s evolving needs and even creating barriers to usage. However, changes to programming following the renovations may have contributed to the increased usage.

The major urban multipurpose indoor recreational facility was the most used in the intervention region in both years. However, its revitalization did not increase usage (usage demand was over capacity prior to revitalization). Revitalization doubled the size of indoor leisure ice arenas, improved track accessibility, expanded the gymnasium, multipurpose community activity rooms and the indoor playground area and added a wellness centre, fitness spaces and youth lounge. These upgrades may not have addressed some barriers to using this facility, as has occurred elsewhere.^32^ Focus group participants described non-physical environmental factors such as crowdedness and cleanliness as barriers, for instance.^24^

The lack of change in usage in the revitalized urban indoor ice and dry arena may have been because the revitalization plan did not address some barriers. For example, focus group participants were concerned that the facility prioritized hockey-related activities over recreational ice skating.^24^ Demand for ice surfaces that exceeds facility availability is typical for municipalities across Alberta and is further complicated by demand from competing activities.

In both regions, usage of the golf courses decreased over time, possibly due to the high costs of playing golf across North America and the sport’s decreased popularity among younger generations.^33^ We also found decreased usage of off-leash dog parks in the comparison region and no change in usage in the upgraded equivalent spaces in the intervention region.

Outdoor space usage levels in the intervention region did not increase after revitalization. Outdoor spaces (except for golf courses) offer opportunities for recreation at no cost to communities regardless of their socioeconomic status. Parks, multiuse trails and other types of outdoor spaces can be an alternative to indoor recreational facilities, especially for people living at low income who cannot afford membership fees, drop-in rates and sport equipment. The populations of both regions used indoor and outdoor spaces, on average, on a monthly and weekly basis, respectively, at both baseline and follow-up periods. Comparison of the baseline and follow-up usage levels of indoor and outdoor venues revealed no statistical increase. Still, it should not be inferred that the intervention region’s revitalization plan did not succeed in increasing usage. First, this study reports on a relatively short-term follow-up assessment of usage after the revitalization, and our follow-up survey coincided with the end of Phase 1 of the revitalization plan due to construction delays. Second, the 15-year intervention plan and upgrading of the other recreation infrastructure was still underway at the time of our analysis. Assessment of usage after completion of all phases of the plan may describe another scenario. 

Of note, at the time of writing, the intervention region had completed Phase 2 (2014–2018) and Phase 3 (2019–2023) of the revitalization plan, which involved improvements to other existing recreation infrastructure.^34^ Analysis of the impacts of these phases at the population level were not performed due to funding constraints and the nature of the available local data.

However, our findings suggest that strategies targeting only physical infrastructures may not result in increased usage across a municipal population. While the recreational venues are geographically dispersed across the region, which would ostensibly facilitate access, local sociocultural needs and leisure-time PA-associated costs may have precluded more frequent use. As discussed elsewhere,^10^,^11^ a key component in revitalizing physical environments is the creation of new PA programs and activities that attract new users and increase the usage levels of current users.

Our null results may be further explained by population preference. Municipal recreation infrastructure may not appeal to everyone, despite the intention that these resources be inclusive, accessible and community-wide. After Phase 1, our focus groups participants described barriers at the intrapersonal level (e.g. family dynamics); facility features unrelated to the PA infrastructure (e.g. unhealthy food options offered by food vendors); facility programming (e.g. no programs for youth and older adults); economic aspects (e.g. high membership fees); social environment (e.g. safety concerns); and policy environment (e.g. inadequate public transportation to get to recreational spaces).^24^ These factors determined participants’ usage of the facilities, but from a policy perspective, went beyond the scope of the intervention region’s revitalization plan. Therefore, initiatives with the aim of addressing inequities in access to publicly funded, health-promoting community resources should consider both the built and social environments when designing and evaluating interventions.^1^,^35^,^36^ Considering people’s experiences when using or trying to use these venues is key to tailoring the intervention to local needs.^10^,^37^ That is particularly relevant for longer-term interventions conducted in multiple phases because the reassessment of residents’ needs allows for adjusting the ongoing and future revitalization activities vis--vis demographic and social changes.

Recreation literature^6^,^37^ and socioecological frameworks^35^,^36^ call for coordinated, multifaceted population-level interventions that target people’s diverse physical and social needs. A recent meta-narrative evidence synthesis on urban green spaces identified stronger evidence for interventions combining built environment changes and PA programming compared to only built environment–oriented interventions.^1^

Our findings reinforce the need for collaborative multisectoral work around a common, shared agenda for a more effective population health intervention and healthier and more active people. Strengthening existing partnerships between municipal departments (e.g. recreation and leisure service delivery, education) may increase the usage of public recreational venues. For instance, improving travel connections in urban settings may enhance usage of venues^1^ and offering organized activities will likely increase the usage of outdoor spaces.^22^ In rural settings, promoting community trails as social gathering places (e.g. sporting events and charity walks and races) may increase the usage of public outdoor spaces.^38^ A multilevel approach informed by the socioecological framework may more effectively improve usage of public recreational venues.^35^

These findings remain relevant and may be even more critical to the current Canadian context of austerity measures and increased costs of living.^39^,^40^ Engagement of residents to assess community needs and of intersectoral partnerships to address barriers to get to and use the recreational venues for different activities are key to guiding community investments in public recreation infrastructure, especially when public resources are scarce. As more people are struggling financially, providing opportunities to use and enjoy the newly renovated, cost-free outdoor spaces can support PA engagement, socialization and well-being.

***Strengths and limitations***

Limitations of this study include the potential coverage bias that occurs with conducting landline telephone interviews and the relatively low response rate (19% to 33%). Loss to follow-up was also a limitation as only 38.2% of the participants in 2011 completed the questionnaire in 2013. In our sensitivity analysis, we found statistically significant differences between the sample who completed the 2011 survey alone and the sample who completed both surveys, which means that our findings may not be generalizable to the full pre-survey population. The missingness in our dataset was related to item nonresponse (shown in the high percentage of missing income data).

This study did not capture PA engagement; we measured the impact on indoor and outdoor space usage for PA purposes. Because of the construction delays, the follow-up survey was conducted near the end of the Phase 1 of the revitalization plan; study funding constraints precluded conducting the survey later, although the results would probably have been different had the survey been administered when residents had had longer to become aware of the upgrades. The results might also have been different if the data had been collected in winter or spring, but to ensure comparability, both surveys were administered at between the end of summer and the middle of fall. The timing of the revitalization may have played a role in participants’ responses as some spaces were revitalized in 2011 and others in 2013, when only a few months had elapsed between completion of the upgrades and administration of the follow-up survey. 

A full analysis of representativeness for each region was not performed; however, because municipal recreational venues are not uniformly used in the regions, those who participated in the survey either had a positive or negative motivation for self-reporting on this topic.

Another limitation was the impossibility of being able to control multiple external factors affecting our outcome—an inherent disadvantage of quasi-experimental studies. However, the quasi-experimental design provides stronger evidence regarding the degree and the direction of temporal changes,^41^ to better support decision-making and resource allocation. 

To date few longitudinal quasi-experimental studies have examined environmental interventions for PA promotion, and most of the existing ones have focused on outdoor spaces.^13^,^42^ Our study adds to the literature by including both indoor and outdoor spaces and different types of recreation infrastructure; and by analyzing the usage of recreational venues located in both urban and rural settings.

Considering the time delay between the environmental intervention and subsequent impacts on population-level health outcomes,^13^,^41^ an advantage of our study is the use of a 2-year follow-up period instead of the more typical follow-up of less than 1 year.^13^,^42^ Another advantage is the provision of real-time information on the impact of Phase 1. Assessing the Phase 1 of an ongoing strategic plan helped the region evaluate current revitalization activities to better tailor future efforts to meet the goal of increasing usage among residents. The other strengths are the inclusion of both youth and adult populations and collection of pre–post data at the same time of year to control for seasonal variation, which is important for outdoor spaces.^1^

## Conclusion

Our longitudinal analysis with a comparison group found that the Phase 1 goals of a three-phase municipal revitalization plan to increase usage levels of the interconnected major and specialized public indoor and outdoor recreational venues had not been fully realized at the time of the study. Upgrading the physical infrastructure of public recreational venues is fundamental for increasing usage levels; however, revitalization followed by the implementation of strategies to address perceived barriers to accessing venues are more likely to increase usage. We recommend that environmental interventions seek to promote equitable inclusivity, livability and well-being, taking into account residents’ perceptions and experiences of municipal venues. Public recreational venues offer much more than PA opportunities. These community-wide resources act as places of connection, well-being and health promotion. Thus, municipal efforts to create a welcoming environment in public recreational facilities and outdoor spaces may help attract diverse users, from professional and amateur athletes to spontaneous users seeking to get out in their community.

Working with smaller budgets, decision-makers and managers of public recreational venues are seeking alternatives to maximize cost-savings while improving residents’ usage levels. This might require investing in programming and marketing and promotion activities for outreach to ensure residents are aware of and motivated to use the upgraded recreational venues, thus increasing usage among current users and attracting new ones.

Environmental audit tools may provide some context of the physical and working conditions of equipment and amenities and other social aspects of facilities (e.g. trained staff and changes in demand for access to indoor public spaces during extreme weather emergencies). Defining a benchmark of usage for each venue may support the revitalization plans and program development. Finally, collaborative work from multiple departments (e.g. urban design, public health) can offer a systems or wrap-around approach for mitigating barriers faced by residents. Exploring the impacts of the revitalized recreational venues located in urban and rural settings on urban or rural residents, respectively, can support further analysis on the successes of local community investments in meeting the diverse needs of the local population group.

Little is known about the impact of public investments in existing indoor and outdoor recreational spaces,^1^,^7^,^10^ which compromises evidence-informed decision-making by municipal, provincial and federal governments regarding returns on investments with smaller public budgets. Identifying effective population-level environmental strategies to increase usage of these spaces is needed to help guide investments and to serve as a cornerstone for multifaceted, comprehensive population health initiatives.

## Acknowledgements

We thank Strathcona County, the City of Spruce Grove, Parkland County and the Town of Stony Plain for their partnerships during this project; without them this work would not have been possible. We are also grateful to Strathcona County for providing financial support for a summer student to work on the project.

We would like to acknowledge Prairie Research Associates who conducting the telephone survey on behalf of the research team. We also thank the residents of the intervention and comparison regions who took the time to participate in this study. We thank Dr. Tanya Berry (project co-investigator) of the Faculty of Kinesiology, Sport, and Recreation, University of Alberta, for her help in grant writing, data collection and data analysis.

CIJN held an Applied Public Health Chair (2014–2019) from the Canadian Institutes of Health Research in partnership with the Public Health Agency of Canada and Alberta Innovates—Health Solutions (2014–2019; CPP 137909).

## Funding

This work was supported by the Canadian Institutes of Health Research (Funder ID: 112692) and Strathcona County Department of Recreation, Parks and Culture. 

## Conflicts of interest

CIJN is a member of the *HPCDP Journal *Editorial Board, but had no role in the peer review or editorial decision-making for this manuscript.

The authors declare no conflicts of interest.

## Authors’ contributions and statement

APB: Investigation, methodology, visualization, writing—original draft, writing—review and editing.

LN: Data curation, investigation, project administration, writing—review and editing.

VK: Formal analysis, methodology, visualization, writing—review and editing.

CIJN: Conceptualization, data curation, funding acquisition, investigation, methodology, supervision, writing—original draft, writing—review and editing.

The content and views expressed in this article are those of the authors and do not necessarily reflect those of the Government of Canada. 
